# Clinical Characteristics and Prognostic Impact of Short Physical Performance Battery in Hospitalized Patients with Acute Heart Failure—Results of the PROFUND-IC Registry †

**DOI:** 10.3390/jcm12185974

**Published:** 2023-09-14

**Authors:** Lidia López-García, Noel Lorenzo-Villalba, Juan Igor Molina-Puente, Aladin Kishta, Beatriz Sanchez-Sauce, Fernando Aguilar-Rodriguez, Máximo Bernanbeu-Wittel, Nuria Muñoz-Rivas, Llanos Soler-Rangel, Luis Fernández-Carmena, Emmanuel Andrès, Francesco Deodati, Francisco Trapiello-Valbuena, Pilar Casasnovas-Rodríguez, Manuel Lorenzo López-Reboiro, Manuel Méndez-Bailon

**Affiliations:** 1 Facultad de Enfermería, Universidad Complutense, Hospital Clínico San Carlos, 28040 Madrid, Spain; 2 Service de Médecine Interne, Hôpitaux Universitaires de Strasbourg, 67000 Strasbourg, France; 3 Servicio de Medicina Interna, Complejo Hospitalario de Ávila, 05004 Avila, Spain; iggidoc@hotmail.com (J.I.M.-P.); aladinkishta@hotmail.com (A.K.); 4 Servicio de Medicina Interna, Hospital Universitario Fundación de Alcorcón, 28922 Madrid, Spain; 5 Servicio de Medicina Interna, Hospital 12 de Octubre, 29010 Madrid, Spain; 6 Servicio de Medicina interna, Hospital Virgen del Rocio, 41013 Sevilla, Spain; wittel@cica.es; 7 Servicio de Medicina Interna, Hospital Universitario Infanta Leonor, 28031 Madrid, Spain; 8 Servicio de Medicina Interna, Hospital Infanta Sofia, 28702 San Sebastián de los Reyes, Spain; 9 Facultad de Enfermería, Universidad Complutense, 28040 Madrid, Spain; 10 Servicio de Medicina Interna, Hospital Infanta Cristina, 28981 Parla, Spain; 11 Servicio de Medicina Interna, Hospital de Oriente de Asturias, 33011 Castañera, Spain; 12 Servicio de Medicina Interna, Hospital de Torrevieja, 29010 Alicante, Spain; 13 Servicio de Medicina Interna, Hospital Monforte de Lemos, 27400 Lugo, Spain; 14 Servicio de Medicina Interna, Hospital Clínico San Carlos, Instituto de Investigación Sanitaria Hospital Clínico San Carlos (IdISSC), Facultad de Medicina, Universidad Complutense de Madrid, 28040 Madrid, Spain

**Keywords:** heart failure, SPPB, frailty, readmissions, mortality

## Abstract

Background: Most patients diagnosed with heart failure (HF) are older adults with multiple comorbidities. Multipathological patients constitute a population with common characteristics: greater clinical complexity and vulnerability, frailty, mortality, functional deterioration, polypharmacy, and poorer health-related quality of life with more dependency. Objectives: To evaluate the clinical characteristics of hospitalized patients with acute heart failure and to determine the prognosis of patients with acute heart failure according to the Short Physical Performance Battery (SPPB) scale. Methods: Observational, prospective, and multicenter cohort study conducted from September 2020 to May 2022 in patients with acute heart failure as the main diagnosis and NT-ProBNP > 300 pg. The cohort included patients admitted to internal medicine departments in 18 hospitals in Spain. Epidemiological variables, comorbidities, cardiovascular risk factors, cardiovascular history, analytical parameters, and treatment during admission and discharge of the patients were collected. Level of frailty was assessed by the SPPB scale, and dependence, through the Barthel index. A descriptive analysis of all the variables was carried out, expressed as frequencies and percentages. A bivariate analysis of the SPPB was performed based on the score obtained (SPPB ≤ 5 and SPPB > 5). For the overall analysis of mortality, HF mortality, and readmission of patients at 30 days, 6 months, and 1 year, Kaplan–Meier survival curves were used, in which the survival experience among patients with an SPPB > 5 and SPPB ≤ 5 was compared. Results: A total of 482 patients were divided into two groups according to the SPPB with a cut-off point of an SPPB < 5. In the sample, 349 patients (77.7%) had an SPPB ≤ 5 and 100 patients (22.30%) had an SPPB > 5. Females (61%) predominated in the group with an SPPB ≤ 5 and males (61%) in those with an SPPB > 5. The mean age was higher in patients with an SPPB ≤ 5 (85.63 years). Anemia was more frequent in patients with an SPPB ≤ 5 (39.5%) than in patients with an SPPB ≥ 5 (29%). This was also seen with osteoarthritis (32.7%, *p* = 0.000), diabetes (49.6%, *p* = 0.001), and dyslipidemia (69.6%, *p* = 0.011). Patients with an SPPB score > 5 had a Barthel index < 60 in only 4% (*n* = 4) of cases; the remainder of the patients (96%, *n* = 96) had a Barthel index > 60. Patients with an SPPB > 5 showed a higher probability of survival at 30 days (*p* = 0.029), 6 months (*p* = 0.031), and 1 year (*p* = 0.007) with (OR = 7.07; 95%CI (1.60–29.80); OR: 3.9; 95%CI (1.30–11.60); OR: 6.01; 95%CI (1.90–18.30)), respectively. No statistically significant differences were obtained in the probability of readmission at 30 days, 6 months, and 1 year (*p* > 0.05). Conclusions: Patients admitted with acute heart failure showed a high frequency of frailty as assessed by the SPPB. Patients with an SPPB ≤ 5 had greater comorbidities and greater functional limitations than patients with an SPPB > 5. Patients with heart failure and a Barthel index > 60 frequently presented an SPPB < 5. In daily clinical practice, priority should be given to performing the SPPB in patients with a Barthel index > 60 to assess frailty. Patients with an SPPB ≤ 5 had a higher risk of mortality at 30 days, 6 months, and 1 year than patients with an SPPB ≤ 5. The SPPB is a valid tool for identifying frailty in acute heart failure patients and predicting 30-day, 6-month, and 1-year mortality.

## 1. Introduction

Most patients diagnosed with heart failure (HF) are older adults with multiple comorbidities [1]. Patients with multiple comorbid conditions constitute a population with common characteristics: greater clinical complexity and vulnerability, frailty, mortality, functional deterioration, polypharmacy, and poorer health-related quality of life with more dependency [2,3]. The management of the different comorbidities plays a fundamental role in HF since they may be involved in the development of HF, contribute to disease progression, or be associated with a worse prognosis [4].

HF patients usually have several associated comorbidities, and in fact, the absence of these is exceptional. However, most HF registries tend to collect comorbidities associated only with a cardiovascular etiology such as arterial hypertension, diabetes mellitus, hypercholesterolemia, smoking, and atrial fibrillation. These registries ignore other comorbid processes which, although apparently unrelated to the HF process, can have a direct influence on the prognosis of these patients and are often the cause of hospital admission [5]. In the Registro Insuficiencia Cardiaca Aguda (RICA) study [6], which was carried out in Spain to analyze the characteristics of patients admitted for HF to Spanish hospitals and their clinical evolution after hospital discharge, a high prevalence of functional deterioration and severe dependency was observed in 55.9% of patients.

In the hospital setting, frailty is a clinical syndrome that is defined as “a state of increased vulnerability as a result of decreased physiologic reserve and function in multiple systems, with consequent compromise in the ability to cope with any stressful situation” [7,8]. Frailty is often described as being synonymous with dependency, comorbidity, or advanced old age, and although these sometimes accompany frailty, a distinction must be made between them [9,10,11]. Comorbidity, dependence, and old age do not necessarily imply frailty. Although the opposite is usually true, a patient can be frail without multiple comorbidities or dependence for basic activities of daily living. Therefore, we must understand frailty as a series of multidimensional deficits that define a situation of vulnerability in the individual [12]. Elderly patients with HF often have coexisting frailty.

A recent study evaluating the prevalence of frailty in patients with heart failure concluded that 50% of hospitalized older patients with HF were frail. Frailty is associated with higher rates of adverse events, increased risk of hospitalization, and poor long-term survival [11,13,14,15]. However, its frequency and prognosis are not completely defined in the HF patient [9].

A frailty scale that has become one of the most promising tools for the assessment of functional capacity is the Short Physical Performance Battery (SPPB) [16,17]. The SPPB is a valid, reliable, and well-established tool for measuring the degree of patient frailty through functional performance [18,19]. The SPPB score has been found to be related to quality of life and the prevalence of falls in older adults. The SPPB uses movements that mimic basic activities of daily living, functions which are essential and important for independent living [20,21]. This scale has been used in the geriatric patient for many years, but there is still relatively little experience in its use and applicability in patients with HF.

The aim of this study was to determine the clinical characteristics of patients admitted for acute HF and their prognosis according to the SPPB.

## 2. Methods

We conducted a prospective observational multicenter cohort study based on the PROFUND-IC registry of the Spanish Society of Internal Medicine (SEMI). We selected patients admitted for acute heart failure as the main diagnosis in the internal medicine services of 18 hospitals in Spain between September 2020 and May 2022. Inclusion criteria were age > 18 years, main diagnosis of acute HF, and NT-proBNP on admission > 300 pg/mL. Patients with active COVID-19 infection were excluded.

The data were collected prospectively in the internal medicine and cardiology departments from one of the 18 hospitals that collaborated in the admission of the patients (first 24–48 h). Demographic variables were recorded such as sex and age and clinical variables such as HF etiology (hypertensive, ischemic, dilated cardiomyopathy, valvular, or amyloidosis), comorbidities, toxic habits (smoking, alcohol), and Barthel index were recorded. Analytical variables included pro-BNP, hemoglobin, lymphocyte count, creatinine, serum sodium, serum potassium, total cholesterol, LDL cholesterol, albumin, urinary sodium, and potassium. The treatments received during admission were collected, including intravenous iron, red blood cell transfusion, antibiotics, bronchodilators, corticoids, hypertonic saline solution, maximum dose of furosemide during, protein supplements, and treatment at discharge.

Frailty is the main variable to be measured in this study. The tool selected to measure frailty in patients with acute heart failure is the SPPB which consists of three tests:

- three balancing positions with increasing difficulty, each held for 10 s;
- walking 4 m at the usual pace to assess walking speed;
- getting up from a chair without assistance and time to perform five repetitions.

Each test is scored from 0 to 4 points, using previously defined and validated cut-off points, after which a final score of between 0 and 12 points is obtained, with the total score indicating overall physical function. The SPPB was categorized according to the latest studies for analysis into very low (0–3 points), low (4–6 points), moderate (7–9 points), and high (10–12 points) [22]. Frailty assessment of patients by the SPPB was always performed within the first 48 h of admission.

Clinical assessment on admission by the SPPB consisted of the following:

- Feet together: less than 10 s (0 points) or 10 s (1 point);
- Semi-tandem: less than 10 s (0 points) or 10 s (1 point);
- Tandem: less than 3 s (0 points), 3 < 10 s (1 point), or 10 s (2 points);
- Time spent walking 4 m at normal pace: cannot (0 points), more than 8.7 s (1 point), 6.2–8.7 s (2 points), 4.8–6.2 (3 points), or less than 4.8 (4 points);
- Crossing arms across chest and rising from chair: unable (0 points) or able (1 point);
- Five repetitions of previous tests: more than 60 s or cannot (0 points), more than 16.7 s (1 point), 13.7 to less than 16.7 (2 points), 11.2 to less than 13.7 (3 points), or 11.19 s or less (4 points).

The patients were divided into two groups according to an SPPB ≥ 5 and SPPB < 5. All patients were followed up at 30 days, 6 months, and 1 year after hospital discharge and the following variables were collected: readmission, cause of readmission (heart failure/other), exits, or death due to cardiovascular causes.

A descriptive analysis of the variables was performed, and an analysis of the frequencies and percentages is presented. Continuous variables are expressed as means with standard deviation or medians with interquartile ranges (25th to 75th percentiles). A bivariate analysis of the SPPB was performed and, depending on the score obtained, patients were stratified according to an SPPB > 5 and SPPB ≤ 5. After stratification, a description of the variables for each of the groups was made and presented by frequency distribution, mean, and median.

To obtain the possible differences between groups, the Student’s *t*-test and Mann–Whitney U test were used for quantitative variables for distributions that did not meet the normality criteria. For qualitative variables, the Chi-square test (χ^2^) or Fisher’s test was used. Kaplan–Meier analysis was performed for 30-day, 6-month, and 1-year mortality among patients with an SPPB > 5 and ≤ 5. A binary logistic regression analysis adjusted for comorbidities was performed.

Data analysis was performed using the Statistical Package for Social Sciences (SPSS) version 25.0 for Mac OS.

## 3. Results

A total of 482 patients were included with a main diagnosis of acute heart failure, including 269 women (55.8%). The mean age was 82.8 ± 9.3 years, and women were older (83.9 ± 8.5) compared to men. Among the most frequent comorbidities were arterial hypertension (91.1%), atrial fibrillation (62.2%), dyslipidemia (67.9%), chronic kidney disease (52.7%), diabetes mellitus (46.9%), chronic anemia (36.9%), ischemic heart disease (34.9%), chronic osteoarticular disease (27.6%), COPD (22%), diabetes mellitus with retinopathy and symptomatic neuropathy (18.7%), and previous stroke (17.8%). Smoking was reported in 33.7% and alcohol intake in 9%. More than half of the patients (55%) were NYHA II, followed by NYHA III (35.5%), NYHA I (6.8%), and NYHA IV (2.3%). The mean daily dose of furosemide was 113.9 ± 95.7 mg/day (Supplementary Table S1).

Of the 482 patients in the sample, the SPPB was assessed in 449 patients. A total of 77.7% of the patients obtained an SPPB score ≤ 5, while 22.3% of the patients obtained an SPPB score > 5. The categorized SPPB results showed that 60.6% of the patients obtained a very low score (0–3), 22.1% of the patients obtained a low score (4–6), 11.1%, a medium score (7–9), and 6%, a high score (10–12) (Tables S1 and S2).

Patients were divided into two groups according to the SPPB with a cut-off point of an SPPB ≤ 5 (*n* = 349, 77.7%) and SPPB > 5 (*n* = 100, 22.3%). Most patients (61%) with an SPPB ≤ 5 were female and most patients (61%) with an SPPB > 5 were male. The mean age of the patients was higher in patients with an SPPB ≤ 5 (85.6 years). Of note, a higher frequency of certain comorbidities was identified in patients with an SPPB ≤ 5 (*p* < 0.05). Neurological disease with motor deficit and cognitive deficit was found in 8.6% and 13.2% in patients with an SPPB > 5 and SPPB ≤ 5, respectively (*p* = 0.000). Anemia was more common in patients with an SPPB ≤ 5 (39.5%) than in patients with an SPPB > 5 (29%). Similarly, osteoarticular disease was more frequently described in patients with an SPPB ≤ 5 (32.7%) than in patients with an SPPB > 5 (14%) (*p* = 0.000). Diabetes and dyslipidemia were more common in patients with an SPPB ≤ 5 (49.6% and 69.6%, respectively) (*p* = 0.001, *p* = 0.011). In contrast, patients with an SPPB > 5 showed higher frequency in variable smoking and alcohol intake than patients with an SPPB ≤ 5, and 51% smoking and 20% alcohol intake were described in patients with SPPB ≥ 5 (*p* = 0.000) (Table 1).

In relation to the etiology of HF, we found that patients with an SPPB ≤ 5 present with a higher frequency of HF of hypertensive cause (45.2%), showing significant differences with patients with an SBPP > 5 (33.3%) (*p* = 0.017). In contrast, a higher frequency of dilated cardiomyopathy HF was observed in patients with an SPPB > 5, described in 5.1% (*p* = 0.016). The presence of valvulopathies and the number of admissions recorded in the last year were similar in both groups, with no statistically significant differences. In relation to the assessment of functional class according to the NYHA, although no differences were found between groups in the initial stages (in which there were no functional limitations for activity or these were slight) (*p* > 0.05), differences were found between both groups (SPPB ≤ 5 and SPPB > 5) when the functional limitation was moderate (NYHA III) (*p* = 0.001) (Table 1).

During admission, the treatments received by both groups were very similar. There were no statistically significant differences in most of the drugs (*p* > 0.05) except for the administration of hypertonic saline, for which its use was described in the patients with an SPPB ≤ 5 (2.33%) (*p* = 0.002). With respect to the administration of morphine, differences between groups were also identified, with its application being more frequent in patients with an SPPB ≤ 5 (15.36%) than in patients with an SPPB > 5 (4.12%) (*p* = 0.001) (Table 1).

At discharge, there were no differences between the two groups in antihypertensive, lipid-lowering, or diuretic treatment (*p* > 0.05). There were differences (*p* = 0.048) in the use of new anticoagulants, with less administration in patients with an SPPB ≤ 5 (23.2%) than in patients with an SPPB > 5 (33%) (Table 2).

In this cohort, nearly half (43.6%, *n* = 152) of the patients with an SPPB ≤ 5 had a Barthel index < 60, which shows dependence for daily life activities, and 56.4% of patients with an SPPB ≤ 5 (56.4%, *n* = 197) had a Barthel index > 60, correlating with independence for basic activities of daily living. Patients with an SPPB > 5 score had a Barthel index < 60 in only 4% (*n* = 4) of the cases; the remaining patients were rated as independent. Statistically significant differences between groups were seen (*p* = 0.000).

Spearman’s correlation analysis showed a positive correlation (0.728) between the SPPB and Barthel index variables (*p* = 0.000). In-hospital mortality in our total sample of patients was 6.33% (*n* = 28). All deceased patients had an SPPB score ≤ 5 (*p* = 0.000) and 4.98% (*n* = 22) died from HF (*p* = 0.000).

At the 30-day follow-up after hospital discharge, readmission of patients with an SPPB ≤ 5 occurred in 18.54% (*n* = 61). A total of 5.16% were readmitted for HF exacerbation. In patients with an SPPB > 5, hospital readmission for all diagnoses was 3.65% (*n* = 12) and 1.21% (*n* = 4) readmission for HF. At the 6-month follow-up, readmission of patients with an SPPB ≤ 5 was 21.16% (*n* = 102) and 7.08% (*n* = 34) were readmitted for HF exacerbation. At the 1-year follow-up after hospital discharge, 25.52% (*n* = 123) of patients with an SPPB ≤ 5 were readmitted to the hospital and 8.08% (*n* = 39) were readmitted for HF exacerbation (*p* = 0.000). In patients with an SPPB > 5 score, we noted a hospital readmission of 5.8% (*n* = 28) of patients with readmission for HF in 2.90% (*n* = 14).

The 30-day mortality observed according to the SPPB also showed differences in the patient cohorts (*p* = 0.000). Mortality for patients with an SPPB ≤ 5 was 14.85% (*n* = 54) and 7.9% (*n* = 30) died from HF. Mortality for patients with an SPPB > 5 was 0.55% (*n* = 2); both cases were due to HF. The 6-month mortality observed according to the SPPB also showed differences in the patient cohorts (*p* = 0.000). Mortality in patients with an SPPB ≤ 5 was 14.33% (*n* = 69), with 9.55% (*n* = 46) mortality related to HF. The percentage of patients with an SPPB > 5 who died was 0.83% (*n* = 4), all due to HF (*p* = 0.000). The 1-year mortality observed according to the SPPB also shows differences in the patient cohorts (*p* = 0.000). Patients with an SPPB ≤ 5 died in 16.3% (*n* = 79) and 11.5% died from HF (*n* = 55). The percentage of patients with an SPPB ≥ 5 who died was 0.83% due to HF (Table 3).

**Figure 1:**
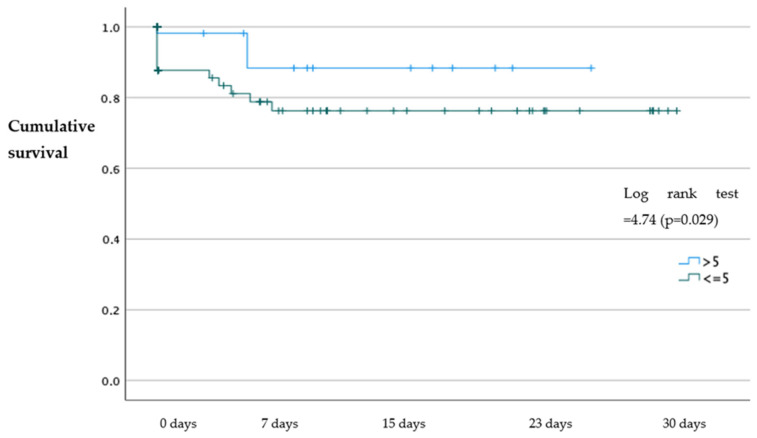
Kaplan–Meier survival analysis of patients with an SPPB ≤ 5 and SPPB > 5 at 30 days.

**Figure 2:**
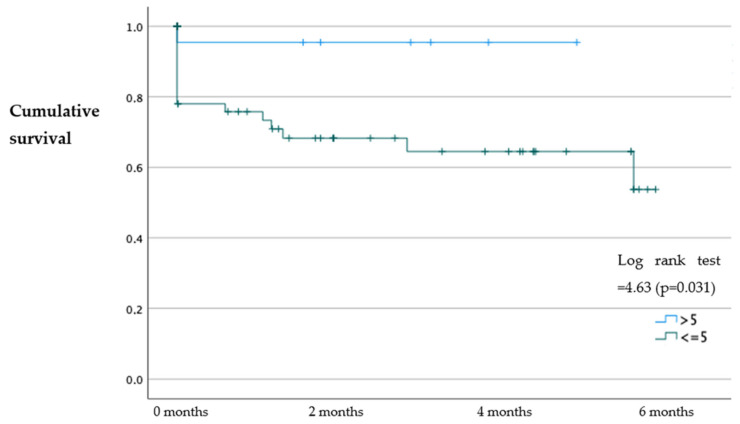
Kaplan–Meier survival analysis of patients with an SPPB ≤ 5 and SPPB > 5 at 6 months.

**Figure 3:**
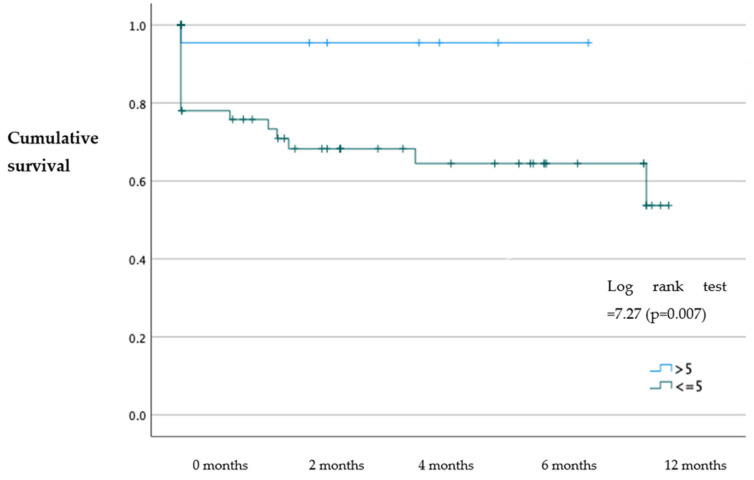
Kaplan–Meier survival analysis of patients with an SPPB ≤ 5 and SPPB > 5 at one year.

The results of our study showed that patients with an SPPB > 5 had a higher probability of survival at 30 days (*p* = 0.029), 6 months (*p* = 0.031), and 1 year (*p* = 0.007) with an OR = 7.07; 95%CI (1.60–29.80); OR: 3.9; 95%CI (1.30–11.60); and OR: 6.01; 95%CI (1.90–18.30), respectively, compared to patients with an SPPB ≤ 5 (Figure 1, Figure 2 and Figure 3). No statistically significant differences were identified in the probability of readmission at 30 days, 6 months, and 1 year (*p* > 0.05).

The initial results obtained after a binary logistic regression for the analysis of the impact of the most frequent and significant comorbidities in patients with an SPPB score ≤ 5 on readmission at 30 days, 6 months, and 1 year were not significant (Table S3).

## 4. Discussion

In our study, we identified the frailty in 77.7% (*n* = 349) of patients hospitalized for acute HF. This was higher than the scores published by other authors. For example, Sze et al. reported frailty rates from 30 to 52% in HF patients, perhaps because of a different tool used [23].

This study emphasizes the assertion that patients with HF should be assessed for the existence of frailty. Decompensations in frail patients with HF are frequent, the clinical manifestations are sometimes non-specific, and the treatments described for HF patients are less effective or more complex to apply. The identification of these patients will help us to initiate multidisciplinary care that improves prognoses, personalizes care, and performs a closer follow-up after hospital discharge. The tool selected to assess the frailty of the patients in this study was the Short Physical Performance Battery (SPPB), in which a score of less than five (SPPB ≤ 5) classified frail patients and a score of more than five (SPPB > 5) classified non-frail patients.

The mean age was higher in patients with an SPPB ≤ 5 (85.6 years) (*p* = 0.000), consistent with other authors who concluded that frailty is a state associated with aging [10,24]. Goldwater and Pinney suggested the need to go a step further and define primary frailty as that produced by aging versus frailty secondary to HF [25]. We believe that, although at this time, it is not possible to clinically differentiate between the two, it is necessary to specify that the frail patient is frail not only because of age but also because of the impact of HF. This helps to understand the interaction between aging, heart failure, and frailty and to guide complex decision-making that affects the elderly patient with a diagnosis of HF. Our study demonstrates that the presence of frailty through the performance of the SPPB in elderly patients with heart failure is very frequent and that the SPPB should be implemented as a routine care tool in the comprehensive assessment of patients with HF.

In the analysis of comorbidities according to the score obtained by the SPPB, we identified a higher frequency of certain comorbidities in patients with an SPPB ≤ 5 (*p* < 0.05). The percentage of patients with neurological disease with motor and cognitive deficits in frail patients was higher than that observed in patients with an SPPB score > 5. These results are similar to those described by other studies showing an association between frailty, cognitive impairment, and motor deficit [26,27,28]. Sometimes, motor deficits manifest as falls, which constitute a major problem due to their high prevalence inside and outside the hospital setting [29].

Atrial fibrillation was more common in patients with an SPPB ≤ 5. This rhythm disorder is frequent in frail and elderly patients and for which there is poor treatment success since frequency control and anticoagulation depend on the characteristics of each patient and his or her circumstances. The difficulty in the control of these patients often precipitates hospital admission, with the resulting increase in their own fragility [24,30,31]. In this regard, in our study, we observed that the most fragile patients with atrial fibrillation receive less oral anticoagulant therapy, an aspect that we should perhaps improve or record in future research to evaluate the efficacy and safety of these treatments in the frail elderly patient with heart failure and atrial fibrillation.

Regarding the analytical parameters, we highlight that anemia was more frequent in the patients with an SPPB ≤ 5 than in the patients with an SPPB > 5 (29%). The development of anemia has been identified as a contributing factor to frailty syndrome. In the FRADEA study [32] conducted in Spain in 2020, the authors concluded that anemia increases the risk of mortality associated with frailty in older adults. This fact implies that the detection, diagnosis, and treatment of this potentially reversible comorbidity could increase their survival. The efficacy of intravenous iron therapy has been demonstrated in patients with HF and iron deficiency (AFFIRM trial) [33]. We should consider this type of therapy in the frail patient with iron deficiency anemia and HF and evaluate its impact on functionality, readmission, survival, and quality of life.

In our study, we found differences between subgroups in which the use of the new anticoagulants was lower in the group of frail patients. It is common in published studies to find benefits derived from treatment with direct-acting anticoagulants versus vitamin K antagonists without consideration of frailty, dependence, or other associated comorbidities that influence the physician when initiating or not initiating anticoagulant treatment [34].

Considering the etiology of HF, we found that patients with an SPPB score ≤ 5 presented a higher proportion of HF of hypertensive etiology than patients with an SPPB > 5. Since the Framingham Study, published by Mahmood et al. in 2014, antihypertensive treatment in these patients has not been discussed. However, the diagnosis, treatment, and control of blood pressure has undergone many changes and controversies in the last decades. Still, it is common that some patients are neither diagnosed nor treated given that hypertension is a totally silent entity. This results in HF of hypertensive origin being more common in older patients [35].

In the functional assessment of HF, we found statistically significant differences between groups (NYHA III), with greater limitation for basic activities of daily living in the SPPB ≤ 5 subgroup. This result agrees with what has been reported in other studies, in which a moderate activity limitation leads to a lack of physical activity and a loss of muscle mass and function. These concepts define sarcopenia, key in the cycle of frailty itself, in which the patient becomes increasingly frail [36,37,38]. It would be interesting in future studies to evaluate the impact of the SPPB on the quality of life of patients admitted for HF.

During patient admission, the treatments received by both groups did not result in statistically significant differences. No differences in diuretic treatment were found between groups, with the dose of furosemide administered being similar in the frail and non-frail patients, despite the finding that the etiology of HF in a patient with an SPPB ≤ 5 tends to be more congestive. As for the administration of hypertonic saline, in this study, it was only described in patients with an SPPB ≤ 5, showing differences with the SPPB > 5 group. According to the latest experiences published in the JACC-HF regarding the use of hypertonic saline in patients with HF, the authors concluded that it is an effective and safe strategy in patients with advanced HF with diuretic resistance [39]. The use of opioids in patients with HF has been part of the traditional treatment for reduction in dyspnea.

In our cohort of patients, dyspnea (NYHA III) was more common in the frail patient. This association between the SPPB and the NYHA functional class has been evaluated in some recent studies. A meta-analysis published by Fuentes et al. in 2020 showed that loss of walking function or reduced walking speed was associated with a higher risk of all-cause mortality compared to patients with heart failure who maintained an adequate walking speed. That study further concluded that for each unit improvement in SPPB, the combined risk of hospitalization and mortality could be reduced [40].

Currently, the use of morphine in patients with HF is controversial; according to a recent study published by Domínguez et al. in 2022, the use of opioids should only be used in palliative care situations and not in the usual treatment of acute heart failure [41,42]. However, morphine and treatment with hypertonic saline and IV furosemide defines a subgroup of patients with more fragile HF and more advanced HF. Given the characteristics, comorbidities, and functional deterioration of frailty patients in our sample and the prognosis of the patients at the 1-year follow-up, we consider that in many cases, these patients would have had advanced or end-stage heart failure.

Our results show a positive correlation between the SPPB and Barthel index variables. However, despite the positive correlation between variables, it should not be assumed that a normal Barthel index score rules out frailty in the patient. The Barthel index discriminates very well against dependence, but not against frailty. As can be seen in the results, more than half of the patients with an SPPB ≤ 5 are considered independent, but they are still frail with all that this entails at the prognostic level. This aspect of our study seems clinically relevant, since we can recommend from the results that patients with HF and a Barthel index greater than 60 who are admitted to the hospital should undergo a frailty assessment by SPPB.

Patients with an SPPB ≤ 5 presented higher mortality during follow-up and higher readmission. We believe that the difference observed in hospital readmission between patients with an SPPB ≤ 5 and SPPB ≥ 5 is considerable and that comorbidities play an important role, since, as has been described, they are more frequent in frail patients. The survival and mortality risk function analysis of our study showed that patients with an SPPB > 5 had longer survival and that patients with an SPPB ≤ 5 had a higher risk of mortality at 30 days, 6 months, and 1 year compared to patients with an SPPB > 5. HF mortality observed by Martinez Santos et al. in 2019 in Spain was 9.2% and increased to 14.5% at the 1-year follow-up. Volpato et al. reached the same conclusion in a multivariable analysis of survival of patients with low SPPB scores upon hospital discharge. These patients had an increased risk of hospitalization and death compared to those with better SPPB scores [43]. In this sense, it is important to detect frailty in the elderly HF patient as it implies a poorer prognosis in the short and medium terms. The prescription of exercise and nutritional support can improve the frailty and prognosis of HF patients. Thus, the intervention suggested by Pacho et al. could be applied to reduce readmissions for CHF and associated mortality in elderly, frail patients with multiple pathologies [44,45].

In contrast, no differences were found in the probability of readmission at 30 days, 6 months, and 1 year. Readmission is a variable influenced after hospital discharge by many factors such as follow-up, early visit, or whether they are referred to an HF unit, internal medicine, or cardiology.

This study has some limitations. The frailty assessment was performed during admission, this being the time of greatest risk, and we did not further evaluate the SPPB during follow-up after patient discharge. In addition, we did not compare the SPPB with other simpler scales such as the Frail scale. Finally, we do not know the impact of the interventions performed in frail patients from a rehabilitatitive point of view because they were not evaluated, and we do not know how they may have influenced prognoses. More research is needed in this regard.

## 5. Conclusions

Patients admitted with acute heart failure showed a high frequency of frailty as assessed by the SPPB. Patients with an SPPB ≤ 5 had greater comorbidities and greater functional limitations than patients with an SPPB > 5. Patients with heart failure and a Barthel index > 60 frequently presented an SPPB < 5. In daily clinical practice, priority should be given to performing the SPPB in patients with Barthel index > 60 to assess frailty. Patients with an SPPB ≤ 5 had a higher risk of mortality at 30 days, 6 months, and 1 year than patients with an SPPB ≤ 5. The SPPB is a valid tool for identifying frailty in acute heart failure patients and predicting mortality at 30 days, 6 months, and 1 year.

## Figures and Tables

**Table 1:** General characteristics of the population studied according to the SPPB.

Variable	SPPB ≤ 5 (*n* (%))	SPPB > 5 (*n* (%))	*p*-Value
Male	136 (39.0)	61 (61.0)	<0.001
Female	213 (61.0)	39 (39.0)	<0.001
Age (mean and SD)	85.63 ± 9.3	81.12 ± 4.5	<0.001
Comorbidity			
Ischemic cardiopathy	121 (34.7)	36 (36.0)	0.596
Vasculitis and systemic autoimmune diseases	16 (4.6)	5 (5.0)	0.567
Chronic renal disease	185 (53.0)	53 (53.0)	0.499
Chronic obstructive pulmonary disease	69 (19.8)	28 (28.0)	0.951
Chronic inflammatory bowel disease	4 (1.1)	2 (2)	0.712
Chronic liver disease	22 (6.3)	8 (8.0)	0.713
Previous cerebrovascular attack	63 (18.1)	14 (14.0)	0.157
Neurological disease with permanent motor deficit	30 (8.6)	1 (1.0)	<0.001
Neurological disease with permanent motor deficit	46 (13.2)	1 (1.0)	<0.001
Symptomatic peripheral artery disease	27 (7.7)	14 (14.0)	0.952
Diabetes mellitus with proliferative retinopathy or symptomatic neuropathy	70 (20.1)	13 (13.0)	0.384
Chronic anemia	138 (39.5)	29 (29.0)	0.022
Active hematologic or other neoplasia	32 (9.2)	9 (9.2)	0.479
Chronic osteoarticular disease	114 (32.7)	14 (14.0)	<0.001
Arterial hypertension	318 (91.4)	90 (90.0)	0.369
Atrial fibrillation	280 (80.9)	58 (59.1)	<0.001
Diabetes mellitus	173 (49.6)	33 (33.0)	0.001
Dyslipidemia	243 (69.6)	57 (57.0)	0.011
Tobacco abuse (ex/active)	99 (28.5)	51 (51)	<0.001
Alcoholism (ex/active)	19 (5)	20 (55)	<0.001
Heart failure etiology			
Hypertensive	155 (45.2)	33 (33)	0.017
Ischemic	71 (20.7)	22 (22.2)	0.638
Dilated cardiomyopathy	1 (0.3)	5 (5.1)	0.016
Valvular	82 (23.9)	21 (21.2)	0.290
Amyloidosis	7 (2.0)	5 (5.1)	0.903
Admissions/year			
0–2	188 (54.0)	62 (62.0)	62 (62.00)
2–4	136 (39.0)	31 (31.0)	0.064
4 or more	24 (6.8)	7 (7.0)	0.517
NYHA functional class			
I	17 (4.9)	10 (10)	0.943
II	192 (55.3)	60 (60)	0.799
III	157 (45.2)	28 (28)	<0.001
IV	11 (3.2)	2 (2)	0.243
LVEF			
LVEF ≥ 50%	258 (75.4)	58 (59.1)	0.001
LVEF < 50%	104 (30.4)	40 (40.8)	0.969
Treatment during admission			
Intravenous iron	84 (24.4)	29 (29.9)	0.149
Blood transfusion	25 (7.2)	5 (5.1)	0.789
Antibiotics	106 (30.9)	24 (24.7)	0.889
Bronchodilators	140 (40.8)	35 (36.0)	0.803
Corticoids	97 (28.2)	20 (20.6)	0.945
Hypertonic saline solution	8 (2.3)	0 (0.0)	0.002
Furosemide	334 (97.3)	96 (98.9)	0.882
Mean furosemide dose (mg)	115.73	99.27	0.988
Protein supplements	27 (7.8)	8 (8.2)	0.553
Morphine	53 (15.3)	4 (4.1)	<0.001
Mechanical ventilation	16 (4.6)	2 (2.0)	0.080
Cardiac catheterization	9 (2.6)	8 (8.2)	0.973
Valvular replacement	7 (2.0)	2 (2.0)	0.508

Legend: LVEF: left ventricular ejection fraction, NYHA: New York Heart Association.

**Table 2:** Treatment at discharge according to the SPPB.

Pharmacologic Class	SPPB ≤ 5 (*n* (%))	SPPB > 5 (*n* (%))	*p*-Value
ACEI	261 (76.5)	73 (73)	0.602
ARB	73 (21.4)	24 (24.7)	0.252
Sacubitril/Valsartan	24 (7.0)	13 (13.4)	0.444
Betablockers	211 (62.0)	58 (59.7)	0.656
Mineralocorticoids	109 (32.2)	38 (39.5)	0.095
No diuretics	31 (9.1)	12 (12.2)	0.196
(Furosemide-Thiazide)	309 (90.8)	86 (87.7)	0.803
iSGLT2	55 (16.3)	24 (24.7)	0.959
Hypolipidemic	104 (57.7)	35 (62.5)	0.262
Anticoagulation	123 (35.2)	42 (42.0)	0.161
Vitamin K antagonists	39 (11.1)	8 (8.0)	0.957
DOACs	81 (23.2)	33 (33.0)	0.048
Mean drugs per patients	10	11	0.716

Legend: ACEI: angiotensin-converting enzyme inhibitors, ARB: angiotensin II blockers, iSGLT2: sodium–glucose cotransporter-2 inhibitors, DOACS: direct oral anticoagulants.

**Table 3:** Mortality and readmissions at 30 days, 6 months, and 1 year according to the SPPB in HF patients.

Period	SPPB ≤ 5 (*n* (%))	SPPB > 5 (*n* (%))	*p*-Value
At 30 days			
Readmissions	61 (18)	12 (12)	ns *
Readmissions for HF	17 (5.1)	4 (4)	ns *
death	54 (14.8)	2 (2)	<0.05
Death due to heart failure	30 (7.9)	2 (2)	<0.05
At 6 months			
Readmissions	102 (21.1)	26 (26)	ns *
Readmissions for HF	34 (7.0)	13 (13)	ns *
death	69 (14.3)	4 (4)	<0.05
Death due to heart failure	46 (9.5)	4 (4)	<0.05
At 1 year			
Readmissions	123 (25.5)	28 (28)	ns *
Readmissions for HF	39 (8.0)	14 (14)	ns *
Death	79 (16.3)	4 (4)	<0.05
Death due to heart failure	55 (11.4)	4 (4)	<0.05

* ns = not statistically significant.

## Data Availability

The data presented in this study are available on request from the corresponding author. The data are not publicly available due to privacy restrictions.

## Supplementary Materials

The following supporting information can be downloaded at: https://www.mdpi.com/article/10.3390/jcm12185974/s1, Table S1. Frailty assessment through Short Physical Performance Battery (SPPB). Table S2. Description of the sample according to categorized SPPB score. Table S3. Binary logistic regression.
