# Supplementary material for: Clinical Characteristics and Prognostic Impact of Short Physical Performance Battery in Hospitalized Patients with Acute Heart Failure—Results of the PROFUND-IC Registry
Source: J Clin Med. 2023 Sep 14;12(18):5974. doi: 10.3390/jcm12185974 (PMC10531733; doi:10.3390/jcm12185974)
Supplement: Supplementary file 1 [file jcm-12-05974-s001.zip › jcm-2554839-supplementary.pdf]

Table S1. Frailty assessment through Short Physical Performance Battery (SPPB).

POSITION	TIME	[n (%)]
**Feet together**	Less than 10s	199 (44.2)
	10s or more	251 (55.80)
**Semi tandem feet**	Less than 10s	277 (57.5)
	10s or more	163 (37.0)
**Tandem Feet**	Less than 3s	291 (66.3)
	3-9.9 s	90 (20.5)
	10s	58 (13.3)
**Walk 4 meters**	Cannot perform	142 (31.6)
	More than 8.7 sec	135 (30.1)
	6.2-8.7 sec	78 (17.4)
	4.8-6.2 sec	54 (12.0)
	Less than 4.8 sec	40 (8.9)
**Rising from a chair with arms crossed**	Cannot perform	307 (68.4)
	Can perform	142 (31.6)
**Rising from a chair with arm crossed over (5 repetitions)**	More than 60s or unable	31 (21.8)
	More than 16.7 s	46 (32.4)
	13.7-16.6 s	23 (16.2)
	11.2-13.6 s	30 (21.1)
	11.1 s or less	12 (8.5)
SPPB categorization		[n (%)]
**>5 points**		100 (22.3)
**≤5 points**		349 (77.7)

Legend: SPPB; Short Physical Performance Battery.

Table S2. Description of the sample according to categorized SPPB score.

SPPB	[n (%)]
Very low (0-3)	272 (60.6)
Low (4-6)	100 (22.1)
Medium (7-9)	50 (11.1)
High (10-12)	27 (6.0)

Legend: SPPB; Short Physical Performance Battery.

Table S3. Binary logistic regression

**Readmissions at 30 days**		
**VARIABLE**	**Exp (B)**	**P-value**
SPPB≤5	1,42(1,27-1,56)	0,313
**Readmissions at 6 months**		
**VARIABLE**	**Exp (B)**	**P-value**
SPPB≤5	0,49(0,45-0,53)	0,073
**Readmissions at 1 year**		

VARIABLE	Exp (B)	P-value
SPPB≤5	0,71 (0,63-0,78)	0,503
